# When a maternal heterozygous mutation of the *CYP24A1* gene leads to infantile hypercalcemia through a maternal uniparental disomy of chromosome 20

**DOI:** 10.1186/s13039-021-00543-4

**Published:** 2021-05-05

**Authors:** Marguerite Hureaux, Sandra Chantot-Bastaraud, Kévin Cassinari, Edouard Martinez Casado, Ariane Cuny, Thierry Frébourg, Rosa Vargas-Poussou, Anne-Claire Bréhin

**Affiliations:** 1 Département de Génétique, Assistance Publique Hôpitaux de Paris, Hôpital Européen Georges Pompidou, 20 rue Leblanc, 75015 Paris, France; 2 Reference Centre for Hereditary Renal Diseases (MARHEA), Paris, France; 3 Paris Cardiovascular Research Center, INSERM, Paris, France; 4 Assistance Publique-Hôpitaux de Paris, Departement de Genetique Medicale, Hôpital Trousseau, 75012 Paris, France; 5 Normandie Univ, UNIROUEN, Inserm U1245 and Rouen University Hospital, Department of Genetics and Reference Center for Developmental Disorders, Normandy Center for Genomic and Personalized Medicine, 76000 Rouen, France; 6 Department of Pediatrics, Centre Hospitalier Universitaire de Rouen, 76000 Rouen, France

**Keywords:** Uniparental disomy of chromosome 20, Infantile hypercalcemia, *CYP24A1*, Silver-Russell-like syndrome

## Abstract

**Background:**

Infantile hypercalcemia is an autosomal recessive disorder caused either by mutations in the *CYP24A1* gene (20q13.2) or in the *SLC34A1* gene (5q35.3). This disease is characterized by hypercalcemia, hypercalciuria and nephrocalcinosis in paediatric patients.

Maternal uniparental disomy of chromosome 20 [UPD(20)mat], resulting in aberrant expression of imprinted transcripts at the *GNAS* locus, is a poorly characterized condition. UPD(20)mat patients manifest a phenotype similar to that of Silver-Russell syndrome and small for gestational age-short stature.

**Case presentation:**

We report here the genetic and clinical characterization of a male child with a phenotype of infantile hypercalcemia, postnatal growth retardation, and minor dysmorphic features. Genetic analysis using a next generation sequencing panel revealed a homozygous pathogenic variant of *CYP24A1*. The absence of the variant in the father led to microsatellite segregation analysis, suggestive of UPD. SNP-array revealed a large terminal copy neutral loss of heterozygosity leading to *CYP24A1* homozygosity. SNP-array data of parent–child trio confirmed a UPD(20)mat responsible for both infantile hypercalcemia and Silver-Russell syndrome-like traits.

**Conclusion:**

This is the first report of uniparental disomy of chromosome 20 revealed by infantile hypercalcemia related to *CYP24A1* biallelic homozygous variants, underlying the importance of controlling allelic segregation in cases of homozygosity.

**Supplementary Information:**

The online version contains supplementary material available at 10.1186/s13039-021-00543-4.

## Background

Infantile hypercalcemia (IH) is a rare genetic cause of nephrocalcinosis typically occurring in paediatric subjects and characterized by inappropriate increment of calcitriol with persistent hypercalcemia, absorptive hypercalciuria, suppressed parathyroid hormone level and nephrocalcinosis [1]. Some patients remain asymptomatic during infancy and present later in life with recurrent episodes of nephrolithiasis [2].

This condition results from biallelic loss-of-function variations in the *CYP24A1* gene, involved in vitamin D catabolism and in calcium homeostasis (IH type 1; OMIM 143880, 20q13.2) [3], or in the *SLC34A1* gene, encoding for the renal sodium-phosphate transporter NaPi-IIa (IH type 2; OMIM 616963, 5q25.3) [4]. Uniparental disomy (UPD) is the inheritance of both homologous chromosomes of a specific chromosome pair from a single parent with two main subtypes: heterodisomy if the two different homologues chromosomes are transmitted and isodisomy if identical homologues chromosomes are transmitted [5]. UPD has been described for almost all the human chromosomes, and could lead to an abnormal phenotype, particularly if it involves an imprinted region [5]. UPD of chromosome 20 is a rare condition associated with a variable phenotype depending on its parental origin. Paternal uniparental disomy of chromosome 20, that includes the *GNAS* locus, has been identified in about 20 sporadic patients with pseudohypoparathyroidism 1B [6] whereas UPD(20)mat with normal karyotype (Mulchandani-Bhoj-Conlin syndrome, OMIM #617352) has been identified in 20 patients with pre- and post-natal growth failure, severe short stature with proportional head circumference and profound feeding difficulty phenotype [7–10]. Those clinical features overlap with that of Silver-Russell syndrome (SRS) and small for gestational age-short stature (SGA-SS) for which genetic bases are heterogeneous; the most frequent being imprinting anomalies of chromosomes 7 and 11 [11, 12].

In this report, we present the first patient with UPD(20)mat revealed by phenotype of IH related to *CYP24A1.* This exceptional situation in rare metabolic disease with recessive transmission underlines the importance of allelic segregation control in cases of homozygosity to determine the risk of recurrence in siblings.

## Case presentation

**Fig. 1:**
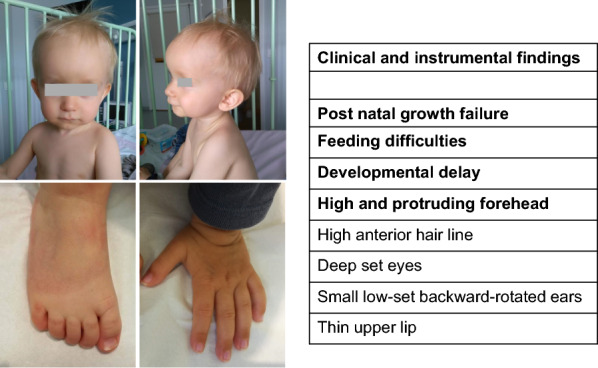
Clinical characterization of the case. Frontal and lateral view of the proband at the age of 14 months. Note the frontal bossing, high anterior hair line, deep set eyes, small low-set backward-rotated ears, and thin upper lip. Other clinical findings are listed in the side table, where 3 out of 6 NH-CSS for SRS are in bold characters

The patient, a 3 years old boy (Fig. 1), is the third child of healthy unrelated French parents. The mother, 45 years old at the time of conception, had two healthy children from a previous union.

The patient was born at a gestational age of 37 weeks with a birth weight of 2750 g (32nd percentile), a birth length of 46 cm (13th percentile) and a birth head circumference of 34 cm (55th percentile). As usually, he had vitamin D prescription (1200 UI of cholecalciferol/day) that he received irregularly during the first 2 months of life. At the age of 9 months, he was referred to the Department of Paediatrics due to growth retardation. Biological tests and clinical examination were normal.

At the age of 12 months, he was evaluated for persistent short stature and developmental delay with feeding difficulties and vomiting. A parental abuse was suspected but feeding difficulties and vomiting persisted during hospitalisation. His weight was 6270 g (− 2.5 SD) and his length was 70.5 cm (− 2.5 SD). On evaluation, there was no evidence for a secondary growth disorder. Except a mild developmental delay, neurological examination was normal. Cardiac ultrasonography and cerebral MRI did not reveal any abnormality. Thyroid function tests and concentrations of IGF-1, calcium, inorganic phosphate and alkaline phosphatase were all within normal limits. There was no evidence of a metabolic disease (Table 1).

**Table 1:** Biological characteristics

	12 months	14 months	18 months	40 months
Plasma [*normes at age*]				
Creatinine (µmol/l) [15–37]	34	25	21	31
CO2 total (mmol/l) [19–24]	17	15	17	24
Potassium (mmol/l) [3.1–4.7]	4.8	4.7	4.2	4
Sodium (mmol/l) [133–140]	137	139	134	141
Magnesium (mmol/l) [0.6–1.3]			0.79	
Chloride (mmol/l) [95–105]	100	100	96	105
Total calcium (mmol/l) [2.20–2.83]	2.54	2.73	2.52	2.54
Ion calcium (mmol/l) [1.22–1.40]				
Phosphate (mmol/l) [1.55–2.39]	**1.27**	**1.4**	**1.15**	**1.52**
PTH (ng/L) [7–31]		** < 4**	**7**	8
25 OH-vitamin D (ng/ml) [50–80]		62	72	63
1,25(OH)_2_-vitamin D (pg/l) [30–150]			**200**	**165**
Urine				
Calcium/creatinin (mmol/l/mmol/l) [< 1.1]			**1.16**	
Sodium (mmol/l)			37	
Phosphate (mmol/l)			11.4	
TRPh (%)			88	

Abnormal values evocative of IH phenotype in bold

Clinical examination suggested a mild facial dysmorphism with a frontal bossing, a high anterior hairline, deep-set eyes, small low-set backward-rotated ears, and a thin upper lip corresponding at 3 out 6 on the Netchine-Harbison clinical scoring system for SRS criteria [11] (Fig. 1). The clinical features of our patient are compared to a cohort of UPD(20)mat reviewed by Hjortshøj et al. [13] (Supplementary Table 1).

After this evaluation, calcium/vitamin D supplementation was resumed.

At the age of 18 months, a renal ultrasonography showed a bilateral nephrocalcinosis grade II, associated to hypercalciuria, hypophosphatemia, increased 1,25 OH vitamin D with abnormally low parathyroid hormone level (PTH) (Table 1).

## Methods of relevant genetic analysis

### Molecular analysis of *CYP24A1*

Genomic DNA was isolated from white blood cells using standard procedures. Next generation sequencing (NGS) was performed using a specifically designed panel for known genes involved in tubulopathies or nephrocalcinosis as previously described [14, 15], and analysed on a Miseq Plateform (Illumina, San Diego, CA). All of the targeted regions were covered at > 30X. Bioinformatics analysis was performed using an in-house pipeline (Polyweb software interface designed by the Paris University Bioinformatics platform) targeting on the region of interest of IH (*CYP24A1* and *SLC34A1* genes). For in silico analysis Alamut V.2.10 software (Interactive Biosoftware, Rouen, France; http://www.interactivebiosoftware.com) was used. All variants identified were confirmed by Sanger sequencing, on a 3730xl DNA Analyzer (Applied Biosystems, ThermoFischer Scientific Waltham, Massachusetts, USA). Variants of interest were classified according the American College of Molecular Genetics guidelines [16].

### Microsatellite analysis

Using Total DNA previously extracted, we performed polymerase chain reaction (PCR) to amplify 5 microsatellite loci located on chromosome 20. (Details of microsatellites used and of amplification protocol in Supplementary data Table 2 and supplementary Methods 1). Microsatellite genotypes were determined by Gene Mapper Software v5.0 (ThermoFischer). PCR was performed several times to confirm genotypes reproducibility.

### SNP-array

Patient and parents were genotyped using HumanOmniExpress-24 microarrays (Illumina, San Diego, CA, USA) which contain up to 710,000 markers. Automated Illumina microarray experiments were performed according to the manufacturer’s instructions. Images were acquired using an iScan System (Illumina). Image analysis and automated Copy Number Variant (CNV) calling were performed using GenomeStudio v.2.0 and CNVPartition v.3.1.6. The Single Nucleotid Polymorphisms (SNP) profiles were analyzed by examination of signal intensity (Log R ratio, i.e. ln (sample copy number/reference copy number)) and allelic composition (BAF, i.e. B Allele Frequency). The Log R ratio can detect CNV and the BAF can estimate the genotype of each SNP: e.g. for an SNP with 2 copies, BAF = 0 means an (AA) genotype, BAF = 0.5 means an (AB) genotype, BAF = 1 means a (BB) genotype.

Results were indicated according to the International System for Human Cytogenomic Nomenclature (ISCN 2016).

## Results

First-line genetic explorations, systematic fragile X syndrome at FRAXA locus and array-CGH (Array comparative genomic hybridization; Agilent 180 K), were normal. Methylation test for the two differentially methylated regions (DMRs) ICR1 and ICR2 regions at chromosome 11p15, the *GRB10-PEG1/MEST* loci at chromosome 7 and the *DLK1-MEG3* locus at chromosome 14q32 were negative excluding main causes of Silver-Russell syndrome.

**Fig. 2:**
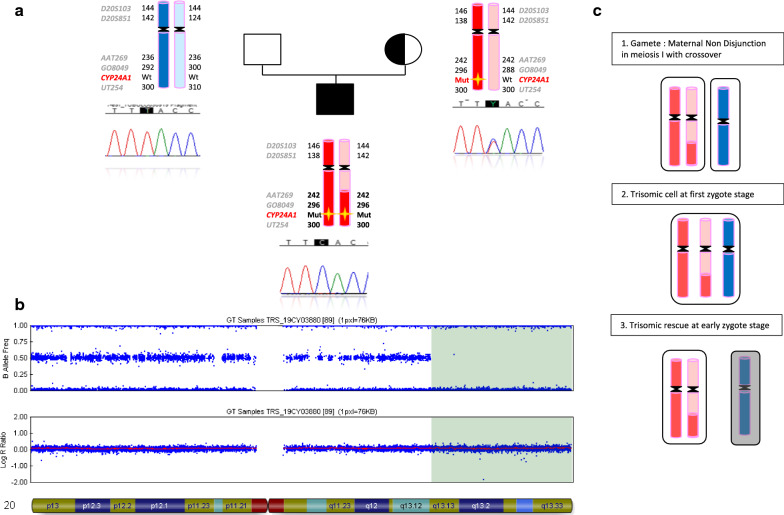
Genetic characterization of UPD(20)mat. (**a**) Family pedigree: filled symbol indicates the homozygous-affected proband and symbol with dot denotes carrier mother. Parents-to-proband segregation of alleles at 6 microsatellites spanning chromosome 20 is shown. Short tandem repeat (STR) markers mapping within the disomic region are highlighted in bold. Chromatograms of parents and proband targeted on the variant genomic position. (**b**) SNP-array profile of patient chromosome 20. Top plot shows B allele frequency revealing an 13.6 Mb isodisomic region (20q13.13-qter) including *SALL4, CYP24A1, MC3R, AURKA, PCK1, VAPB, STX16, GNAS, TUBB1, EDN3, OSBPL2, GATA5, COL9A3, SCLC7A9, RTEL1, CHRNA4, KCNQ2, EEF1A2, PRPF6, NDAJC5* and *SOX18,* imprinted loci; bottom plot shows Log R ratio, which reveals a proper biallelic contribution. (**c**) Estimated mechanism of UPD(20)mat event from gametogenesis to embryo. 1: As a first step, crossing-over during maternal gametogenesis, followed by non-disjunction during Meiosis I; 2: trisomic cell at first zygote stage; 3.trisomic rescue at early zygote stage

NGS analysis identified a homozygous variant in exon 9 of the *CYP24A1* gene: c.[1226 T > C];[(1226 T > C)], p.[(Leu409Ser);(Leu409Ser)]. This variant, previously reported [4], was classified as pathogenic variant (class 5) using the following criteria PS3 PS4, PM2, PP3, PP5 of the ACMG guidelines, described in supplementary Methods [16]. The tool used to evaluate Copy Number Variant integrated to the NGS analysis software showed normal CNV ratios among the whole gene. Parental DNA study showed that the variant was inherited from the mother, who was heterozygous carrier, and that the father was not harbouring the variant. First hypothesis was non-paternity, excluded by analysis of 7 unlinked microsatellite markers located on 6 chromosomes (supplementary Table 2). Further microsatellite analysis targeted on chromosome 20 in 5 loci spread on the two arms 20p and 20q showed that (1) index case was homozygote for AAT269, GO8049, and UT254 located on 20q, (2) haplotype AAT269 (242 bp), GO8049(296 pb), pathogenic variation c.1226 T > C. and UT254 (300 bp) is inherited from the mother, and that (3) D20S103 and D20S853 located on 20p, are present in both father, mother and patient at heterozygous state, that could correspond to different paternal or maternal recombination mechanisms during meiosis. In conclusion, microsatellites segregation showed homozygosity for long arm’s markers AAT269, GO8049, and UT254 markers; and NGS and Sanger sequencing confirmed the homozygous status of the pathogenic variant in *CYP24A1*. Furthermore, lack of paternal contribution for 3 of them suggested uniparental maternal disomy (Fig. 2a).

SNP-array was performed on patient’s DNA in order to test this hypothesis and showed a large terminal homozygous region of 13.6 Mb size without copy loss from 20q13.13 to 20qter including the *CYP24A1* locus (Fig. 2b). This homozygous state only concerned chromosome 20, and therefore was not evocative of consanguinity, according to the ACMG guidelines of 2013 [17] Subsequently, child and parental genotypes determined by SNP-array probes were compared to each other and demonstrated maternal inheritance for the whole 20 chromosome involving combination of heterodisomic and isodisomic region. Such pattern of heterodisomic region including pericentromeric region and isodisomic region is consistent with uniparental disomy subsequent to nondisjunction during meiosis I after meiotic crossover and postzygotic trisomy rescue (Fig. 2b). Child and both parents had normal karyotypes ruling out chromosomal rearrangement prone to 3:1 disjunction with subsequent trisomy rescue (reciprocal translocation between imprinted 20 chromosome and other).

## Discussion

We report here the case of a young boy with UPD(20)mat leading to infantile hypercalcemia. At the first examination, the 12 months boy had post-natal growth retardation with feeding difficulties and vomiting, and facial dysmorphism associated to major growth retardation led to explore main causes of Silver-Russell syndrome. However, methylation analysis of chromosomes 7, and 11 were normal, corresponding to approximately 40 to 60% of known causes of this syndrome [18]. Later, the fortuitous discovery of nephrocalcinosis and hypercalciuria led to suspect an infantile hypercalcemia. NGS sequencing confirmed this diagnosis with the identification of a homozygous pathogenic missense nucleotide variation in the *CYP24A1* gene*.* Parental segregation revealed that the mutation was present in heterozygous state only in the patient’s mother and microsatellites segregation suggested maternal uniparental disomy with isodisomic region neighboring *CYP24A1* locus. This data and the persistence of feeding difficulties and growth retardation despite of cessation of vomiting led to UPD(20)mat suspicion. Trio SNP-array analysis and child’s pattern of heterodisomic and isodisomic region confirmed the diagnosis of maternal disomy of chromosome 20 with isodisomic region generated by meiotic crossover during maternal gametogenesis. As pericentromeric markers showed heterodisomy, a maternal meiosis I error was probably the first step of this UPD formation. As the mother was 45 years old at the time of conception, this observation corroborates the major role of maternal age- in non-disjunction mechanism in meiosis I [19].

Patients with UPD(20)mat mostly present with post-natal growth retardation, small for gestational age, feeding difficulties or low body mass index. In half of cases, developmental delay and hypotonia and slight clinical dysmorphic features can be found [http://cs-tl.de/DB/CA/UPD/0-Start.html [accessed 03/15/2021]]. Only two cases with hetero-isodisomy also present hypercalcemia associated with low PTH, suggestive of IH phenotype; without confirmation of the involvement of the *CYP24A1* gene [7]. In other cases described of UPD(20)mat, no other clinical findings evocative of IH were reported. Beside, IH patients present with hypercalcemia, low PTH, normal to high 1–25 OH vitamin D. Clinically, IH patients present low body mass index or failure to thrive, polyuria or dehydration, hypotonia and nephrocalcinosis at ultrasound analysis [3]. No neurodevelopmental delay has been reported associated to IH.

Symptoms such as growth retardation or feeding difficulties are nonspecific and can have a variety of causes. In our case, the IH phenotype associating hypercalcemia and nephrocalcinosis initially led to the diagnosis of IH and secondarily of UPD(20)mat, in front of a set of arguments such as absence of mutation in the father, persistence of growth retardation and advanced maternal age. Moreover, the patients did not present any other clinical or biochemical anomaly suggestive of other recessive disorder, neither a weak trisomy 20 mosaicism.

In conclusion, this report shows that UPD(20)mat explains the child's phenotype since growth retardation and psychomotor retardation overlaps with SRS features and correspond to elements classically described in Mulchani-Bhoj-Conlin syndrome. To our knowledge, this is the first reported case of UPD(20)mat revealing a deficiency in *CYP24A1*. This exceptional situation in rare diseases of the metabolism with recessive transmission underlines the importance of controlling allelic segregation in cases of homozygosity in order to precisely determine the risk of recurrence in sibling.

## Supplementary Information

**Additional file 1**.

## Data Availability

The authors confirm that the data supporting the findings of this study are available within the article and its supplementary materials.
