# Supplementary material for: When a maternal heterozygous mutation of the CYP24A1 gene leads to infantile hypercalcemia through a maternal uniparental disomy of chromosome 20
Source: Mol Cytogenet. 2021 May 5;14:23. doi: 10.1186/s13039-021-00543-4 (PMC8101107; doi:10.1186/s13039-021-00543-4)
Supplement: Supplementary file 1 — Additional file 1. [file 13039_2021_543_MOESM1_ESM.docx]

**Supplementary data**

**Supplementary Table 1: Phenotype comparison between our patient and 17 patients with UPD(20)mat from Hjortshøj et al.[11]**

|  | **Patient** | **Hjortshoj et al., 2020** |
| --- | --- | --- |
| Age at evalutation | 14 months |  |
| Maternal age (years) | 45 |  |
| **Criteria for SRS phenotype** |  |  |
| Small for Gestational age | - | 15/17 |
| Relative macrocephaly at birth | - | 4/9 |
| Post Natal Growth retardation | **+** | 15/17 |
| Protruding forehead | **+** | 3/8 |
| Feeding difficulties | **+** | 15/16 |
| Body asymetry | - | 1/8 |
| **Other clinical features** |  |  |
| Facial Dysmorphism | **+** | 6/11 |
| Triangular Face | - | 6/8 |
| Hypotonia/ / Developmental delay | **+** | 7/16 |
| Clinodactyly | - | 5/6 |
| Skeletal problems | - | 8/13 |
| Hyperpigmentation | - | 4/7 |
| Low IGF-1 levels | - | 6/11 |

**Supplementary Table 2: Microsatellite and *CYP24A1* analysis**

|  |  | Genotype | | |
| --- | --- | --- | --- | --- |
|  |  | Patient | Father | Mother |
| Chromosome | Marker |  |  |  |
| 1 | D1S199* | 276/276 | 276/276 | 276/276 |
| 1 | D1S243* | 151/156 | 151/156 | 151/156 |
| 2 | D2S389* | 197/209 | 197/209 | 197/199 |
| 3 | D3S1314* | 160/166 | 162/166 | 160/164 |
| 10 | D10S17654* | 172/174 | 174/178 | 172/178 |
| 11 | D11S925* | 170/170 | 170/192 | 170/192 |
| 14 | D14S283* | 122/130 | 130/134 | 122/122 |
| 20 | D20S103 | 146/144 | 144/144 | 146/144 |
| 20 | D20S851 | 138/142 | 142/124 | 138/142 |
| 20 | AAT269 | 242/242 | 236/236 | 242/242 |
| 20 | GO8049 | 296/296 | 292/300 | 296/288 |
| 20 | *CYP24A1* | c.1126T>C;p.L409S hmz | Wt | c.1126T>C;p.L409S htz |
| 20 | UT254 | 300/300 | 300/310 | 300/300 |

Markers with an asterisk [*] correspond to paternity analysis of 7 unlinked microsatellite markers located on chromosomes 1, 2, 3 10, 11 and 14. “hmz” is homozygous status and “htz” is heterozygous status.

Supplementary Methods 1

Microsatellite analysis protocol : PCR was performed with 2 μl of Forward Primer, 2uL of Reverse primer, 1.5 uL of dNTP (2mM), 2.5 uL of buffer (10X), 0.1 uL of polymerase enzyme Taq Qbiogen (Fischer Scientific, New Hampshire, USA), 2 μl of DNA extract (25 ng/μl), and completed with 14.9 uL of water. Thermocycling conditions were 95°C for 5 min; followed by 26 cycles of 95°C for 30 sec, 55°C for 45 sec; and finally 72°C for 7 min.

1 uL of PCR products was then prepared with 20 ul Formamide, 0.5 ul HD400 ROX by wells and after denaturation step by 5 minutes at 95°C, the mix was slightly centrifuged and dive into ice, before sequencing on an 3730xl DNA Analyzer (Applied Biosystems, ThermoFischer Scientific [Waltham, Massachusetts, USA](https://www.google.com/search?client=firefox-b-d&sxsrf=ALeKk00RdTuJg9UY5YuE9f5MxoqaAvmeJA:1587120252132&q=Waltham+(Massachusetts)&stick=H4sIAAAAAAAAAOPgE-LSz9U3MCooMTBJU-IAsTOqjE21tLKTrfTzi9IT8zKrEksy8_NQOFYZqYkphaWJRSWpRcWLWMXDE3NKMhJzFTR8E4uLE5MzSotTS0qKNXewMgIAaSyPy2EAAAA&sa=X&ved=2ahUKEwjFgeDWo-_oAhWRA2MBHdd1ARAQmxMoATARegQIDRAD)).

Supplementary Methods 2

ACMG criteria description used to classify the patient’variant:

| PS: strong evidence of pathogenicity. | PS3: well-established in vitro or in vivo functional studies supportive of a damaging effect on the gene or gene product. |
| --- | --- |
|  | PS4: the prevalence of the variant in affected individuals is significantly increased compared to the prevalence in controls. |
| PM: moderate evidence of pathogenicity. | PM2: absent from controls (or at extremely low frequency if recessive) in gnomAD database. |
| PP: supporting evidence of pathogenicity. | PP3: Multiple lines of computational evidence support a deleterious effect on the gene or gene product. |
|  | PP5: Reputable source recently reports variant as pathogenic but the evidence is not available to the laboratory to perform an independent evaluation. |
